# Reversible Inhibition of Iron Oxide Nanozyme by Guanidine Chloride

**DOI:** 10.3389/fchem.2020.00491

**Published:** 2020-06-11

**Authors:** Wei-chuan Mo, Jia Yu, Li-zeng Gao, Ying Liu, Yan Wei, Rong-qiao He

**Affiliations:** ^1^State Key Laboratory of Brain and Cognitive Sciences, Institute of Biophysics, University of the Chinese Academy of Sciences, CAS, Beijing, China; ^2^CAS Key Laboratory of Mental Health Laboratory, Institute of Psychology, Beijing, China; ^3^School of Life Sciences, Beijing University of Chinese Medicine, Beijing, China; ^4^Southwest Medical University, Luzhou, China; ^5^CAS Engineering Laboratory for Nanozyme, Institute of Biophysics Key Laboratory of Protein and Peptide Drugs, Institute of Biophysics, CAS, Yangzhou, China

**Keywords:** nanozyme, peroxidase, guanidine chloride, activity inhibition, electron spin resonance, g-factor, aggregation

## Abstract

Nanozymes have been widely applied in bio-assays in the field of biotechnology and biomedicines. However, the physicochemical basis of nanozyme catalytic activity remains elusive. To test whether nanozymes exhibit an inactivation effect similar to that of natural enzymes, we used guanidine chloride (GuHCl) to disturb the iron oxide nanozyme (IONzyme) and observed that GuHCl induced IONzyme aggregation and that the peroxidase-like activity of IONzyme significantly decreased in the presence of GuHCl. However, the aggregation appeared to be unrelated to the quick process of inactivation, as GuHCl acted as a reversible inhibitor of IONzyme instead of a solo denaturant. Inhibition kinetic analysis showed that GuHCl binds to IONzyme competitively with H_2_O_2_ but non-competitively with tetramethylbenzidine. In addition, electron spin resonance spectroscopy showed that increasing GuHCl level of GuHCl induced a correlated pattern of changes in the activity and the state of the unpaired electrons of the IONzymes. This result indicates that GuHCl probably directly interacts with the iron atoms of IONzyme and affects the electron density of iron, which may then induce IONzyme inactivation. These findings not only contribute to understanding the essence of nanozyme catalytic activity but also suggest a practically feasible method to regulate the catalytic activity of IONzyme.

## Introduction

Since the discovery of the peroxidase-like activity of iron oxide nanoparticles in 2007 (Gao et al., 2007), the application of nanozymes has rapidly emerged as a novel field. Nanozymes have been widely used in the field of biotechnology and biomedicine, e.g., hydrogen peroxide (H_2_O_2_) detection (Wei and Wang, 2008), DNA detection (Park et al., 2011), and immunohistochemical staining (Wu et al., 2011). To date, nanozymes have been described as nanomaterials with intrinsic enzyme-like activities, which are of broad interest for clinical use (Quick et al., 2008; Kelong et al., 2012; Zhang et al., 2016).

Many researchers believe that the nanoscale effect is a decisive factor in the nanozyme's catalytic activity. Nanomaterials can simulate the function of proteins by modifying their size, surface charge and groups (Kotov, 2010). In particular, the surface charge properties of the nanomaterials are considered to play a key role in the nanoscale effect (Sen and Barisik, 2018). However, it is still lacking direct evidence to support this hypothesis. Given that ferrous ions are capable of catalyzing the degradation of H_2_O_2_ in the Fenton reaction (Fenton, 1894), many researchers believe that the iron atom at the surface of iron oxide nanozymes (IONzyme) probably participates in the Fenton mechanism (Wang et al., 2010; Niu et al., 2011) and contributes to the catalytic capacity. However, the peroxidase-like activity of the non-metal nanozymes (e.g., carbon-based nanozymes) (Song et al., 2010) could not be explained by the Fenton mechanism (Wei and Wang, 2013). Therefore, in addition to the Fenton reaction, other nanozyme mechanisms must be elucidated by further theoretical and experimental studies.

Fan and colleagues simulated the catalytic microenvironment of horseradish peroxidase (HRP) on the surface of IONzyme by histidine residue modification and successfully boosted the catalytic efficiency of IONzyme up to 20.8 times (Fan et al., 2017). This result illuminates another aspect of nanozyme's catalytic activity and showed that protein-based enzymology theory and methodology could facilitate uncovering the underlying mechanism of the enzyme-like activity of nanomaterials. “Enzyme inactivation,” e.g., by site-specific mutation, protein truncation, inhibition, and denaturation to reduce the enzyme activity, is a standard strategy for studying the active site and catalytic mechanism of a protein enzyme by comparing the enzymatic properties of the inactivated protein with those of its native form. Therefore, a controllable method of nanozyme inactivation would add to our understanding of the essence of its catalytic mechanism.

Recently, Zhang and colleagues recorded IONzyme inactivation concurrent with IONzyme aggregation when the IONzymes were suspended in cell culture medium (DMEM with 10% fetal bovine serum). They ascribed the decrease in activity to the medium-induced aggregation of nanoparticles (Zhang et al., 2016). Meanwhile, Liu and colleagues observed that halide ions (Xs) can affect the activity of gold nanozyme. The enzymatic-inhibition effect of Xs did not show time dependence, although the size of the gold nanozymes varied over time (Liu et al., 2017). Although these studies did not reach an agreement on the explanation of nanozyme inactivation, they implied that ion-rich conditions can reduce the enzymatic activity of nanozymes, which further revealed that nanozymes can exhibit inactivation properties analogous to those of protein enzymes. However, an in-depth investigation is still needed to uncover the underlying details.

In this study, we analyzed the effects of GuHCl, a commonly used denaturant for natural enzymes, on the representative peroxidase-like activity of IONzyme in the presence of hydrogen peroxide (H_2_O_2_) and tetramethylbenzidine (TMB). We found that GuHCl induced both aggregation and inactivation of IONzyme and bound to the iron atom on the surface of IONzyme, resulting in inactivation. The substrate H_2_O_2_ may bind to the iron atom because of its competition with GuHCl in binding to IONzyme, and TMB is associated with oxygen atoms because of its non-competition with GuHCl.

## Materials and Methods

### Reagents

Iron-based nanozyme Fe_3_O_4_ magnetic nanoparticles (IONzymes; Diameter, 90 nm) were synthesized according to the solvothermal method (Fan et al., 2017; see the brief description in the materials and methods section in the supporting information). The nanozyme suspensions were ultra-sonicated (250 W, 15 s; SCIENTZ, Zhejiang, China) before use if not stated otherwise. Tetramethylbenzidine chromogen solution (TMB, ε = 3.9 × 10^4^ M^−1^cm^−1^ at 652 nm) was purchased from Wantai Co. Beijing (China). Guanidine hydrochloride (GuHCl) was purchased from Amresco (Solon, OH, USA). Hydrogen peroxide (H_2_O_2_) and sodium acetate buffer (NaAc, pH = 4.5) were purchased from Sinopharm Chemical Reagent (Shanghai, China). Distilled water was retreated and collected on a Thermo Scientific Barnstead Nanopure water purifier (18.2 MΩ; Dubuque, IO, USA).

### Assay of Peroxidase-Like Activity

**Figure 1:**
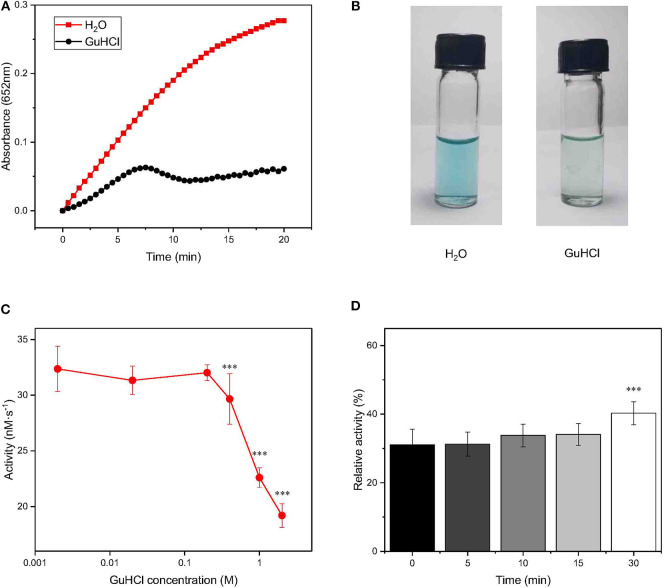
Changes in the activity of IONzyme at different concentrations of GuHCl. IONzyme (final concentration, 0.5 μg/100 μl) was mixed with 2.0 M GuHCl in water at room temperature. H_2_O_2_ (0.6 M) and TMB (0.4 mM) suspended in 200 mM sodium acetate buffer (pH 4.5) were added to the reaction mixture, followed by measurements of the absorbance at 652 nm (pH 4.5, 37°C) **(A)**. Changes in the absorbance between 0 and 150 s were used to calculate the peroxidase-like activity of IONzymes (nM·s^−1^). Aliquots (blue, oxidized TMB) were taken in Eppendorf tubes from the reaction at 2 min, compared with H_2_O as a control **(B)**. IONzyme was mixed with different concentrations of GuHCl, and aliquots were taken for activity measurement (*n* = 12) **(C)**. The data on the relative changes in the peroxidase activity of IONzyme incubated with 2.0 M GuHCl for different times were normalized to those of the untreated control (*n* = 6) **(D)**. Data are the mean ± SD. ***P < 0.001.

The peroxidase-like activities of IONzyme were measured in TMB chromogen solution. IONzyme suspension with or without GuHCl was loaded into a 96-well plate and incubated at room temperature (usually ~2 min unless otherwise stated), followed by the addition of the working solution, and the absorbance (652 nm, see the UV/Vis absorption spectra of reaction solutions containing TMB with or without GuHCl in Figure S3) was recorded at 37°C on a Microplate spectrophotometer (Molecular Devices, Sunnyvale, CA, USA). The working solution containing TMB chromogen solution mixed with NaAc buffer at 1:1 (v/v) with the addition of H_2_O_2_. Given the Nanozyme aggregation probably disturbed the absorption reading (Figure 1A), the initial reaction velocity (*V*_0_) was calculated by the software using 0–300 s reads (Softmax pro 6, Molecular Devices, Sunnyvale, CA, USA) and used to evaluate the peroxidase-like activity of IONzyme. Note that the 100 μL reaction mixture contained concentrations of TMB, H_2_O_2_ and IONzyme of 0.4, 600 mM, and 5 mg/L, respectively, unless otherwise stated. The pH of all the reaction mixtures are 4.5. For the computation of IC_50_, the curves were fit to

(1) $$\begin{array}{l} y=100\%/\left[1+\left(I/{\text{IC}}_{50}\right)\right] \end{array}$$

where *I* is the inhibitor concentration, and *y* is the percentage of activity.

For recycling IONzymes, GuHCl was removed from the reaction mixture by centrifugation (16,000 × g, 25°C, 30 min). After the GuHCl supernatant was discarded, the precipitated IONzyme was washed with H_2_O twice. Then, the washed IONzymes were resuspended in H_2_O or GuHCl to the same volume for the activity assays.

### Inhibition Kinetics Analysis

The inhibition kinetics assays were carried out under the conditions described above, except for measurements of IONzyme activities using different concentrations of TMB at a fixed concentration of H_2_O_2_ (2.7 M) or using different concentrations of H_2_O_2_ at a fixed concentration of TMB (0.4 mM). The apparent kinetic parameters were calculated based on the function

(2) $$\begin{array}{l} V_{0}=V_{max}\times \frac{\left[S\right]}{K_{\text{m}}+\left[S\right]} \end{array}$$

where *V*_0_ is the initial reaction velocity, *V*_max_ is the maximal reaction velocity, [*S*] is the concentration of substrate and *K*_m_ is the Michaelis constant.

### Dynamic Light Scattering (DLS)

The IONzyme was incubated with GuHCl, and the intensities of light scattering were recorded at different time points on a DynaPro NanoStar DLS instrument (Wyatt technology, Santa Barbara, CA, USA). The resulting hydrodynamic radii were calculated according to the manufacturer's instructions.

### Electron Spin Resonance (ESR) Spectroscopy

IONzymes were mixed with NaAC buffer (pH = 4.5) and GuHCl as mentioned above. Then, the mixtures were transferred into a glass capillary and placed in the ESR cavity. All ESR measurements were carried out at room temperature on an ESR spectroscope (Bruker A300-10/12, Billerica, USA) with 20 mW microwave power. The modulation field was 1 G, and the scan range was 6,000 G.

### Statistical Methods

Data are shown as the means ± SD. Data were obtained from at least three independent experiments. One-way ANOVA was applied for mean comparison. Significance was accepted at *p* < 0.05.

## Results

### IONzyme Is Inactivated by GuHCl in a Concentration- and Treatment Time-Dependent Manner

To test whether IONzyme could be inhibited by GuHCl as a natural enzyme could, we mixed IONzyme (ϕ= 90 nm) with GuHCl (2.0 M) and tested the peroxidase activity using a H_2_O_2_-3,3′,5,5′-tetramethylbenzidine (TMB) colorimetric system (Fan et al., 2017). A deeper blue color indicates more oxidized TMB produced. As shown in Figure 1A, the activity of IONzyme, represented by absorbance detection, decreased dramatically in the presence of GuHCl compared with the control group in the absence of GuHCl. The product in the GuHCl-containing system was a shallower blue than that generated in the control, indicating that the enzyme-like activity of IONzymes declined after mixing with GuHCl (Figure 1B).

To reveal the character of IONzyme inactivation by GuHCl, we analyzed the effects with a concentration gradient of GuHCl solution. As shown in Figure 1C, the activity of IONzyme (initial reaction velocity, *V*_0_) significantly decreased at GuHCl concentrations higher than 0.4 M. Higher concentrations gave a stronger effect. The IC_50_ for the decrease in IONzyme activity was ~2.6 M GuHCl. The activity of IONzyme was not markedly affected by GuHCl concentrations no greater than 0.2 M (Figure 1C). When the incubation time of IONzyme with GuHCl increased to 24 h, the GuHCl-induced IONzyme inactivation became stronger within the effective concentrations, and the complete repression of IONzyme was detected in 1 M GuHCl (Figure S1). These data indicated that IONzyme activity can be inhibited by GuHCl in a concentration- and time-dependent manner.

To further investigate the IONzyme-GuHCl interaction, we incubated IONzyme with GuHCl for different lengths of time (0, 5, 10, 15, and 30 min) and measured its activity. As shown in Figure 1D, ~70% decreases in activity could be immediately detected upon the addition of GuHCl. The level of residual activity in the presence of GuHCl did not change when the incubation time was prolonged for no more than 15 min, and the activity fluctuated slightly upon incubation for 30 min. These results indicated that the interaction between GuHCl and IONzyme rapidly approaches equilibrium in the initial stage. The suppression of IONzyme activity may identify GuHCl as an inhibitor of IONzyme.

### GuHCl-Induced Inhibition of IONzyme Is Reversible

**Figure 2:**
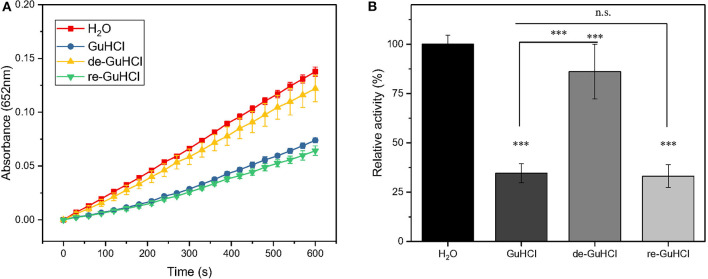
GuHCl is a reversible inhibitor of IONzyme. The peroxidase activity of recycled IONzyme was analyzed and shown by the dynamic changes in the absorbance **(A)** and the relative activity **(B)**. Conditions are shown in Figure 1. Samples included the untreated IONzyme control (H_2_O), IONzyme mixed with GuHCl (GuHCl), recycled IONzyme after the removal of GuHCl (de-GuHCl), and the de-GuHCl sample with GuHCl re-added (re-GuHCl) (*n* ≥ 12). Data are the mean ± SD. ***P < 0.001, No significance (N.S.), *P* > 0.5.

To clarify whether the inhibitory effect of GuHCl on IONzyme peroxidase-like activity is reversible, we tested the peroxidase-like activity of IONzyme recycled from the IONzyme-2 M GuHCl mixture using centrifugation. Upon the addition of GuHCl, As shown in Figure 2, the IONzyme in 2 M GuHCl retained 33% of the activity of the control (*p* < 0.001), and the recycled IONzyme (de-GuHCl sample) exhibited 86% activity. Furthermore, when we re-added 2 M GuHCl to the de-GuHCl sample and tested the activity again, the same level of inhibition compared with the control (~67%) of IONzyme occurred again. The remaining activity showed no significant difference from that of the GuHCl sample before recycling (*P* = 0.9557), and the dynamics curve of the reaction maintained the same pattern (Figure 2B). These data indicate that the inhibitory effect of GuHCl on IONzyme is reversible.

### GuHCl Acts as an IONzyme Inhibitor by Competition With the Substrate H_2_O_2_

**Figure 3:**
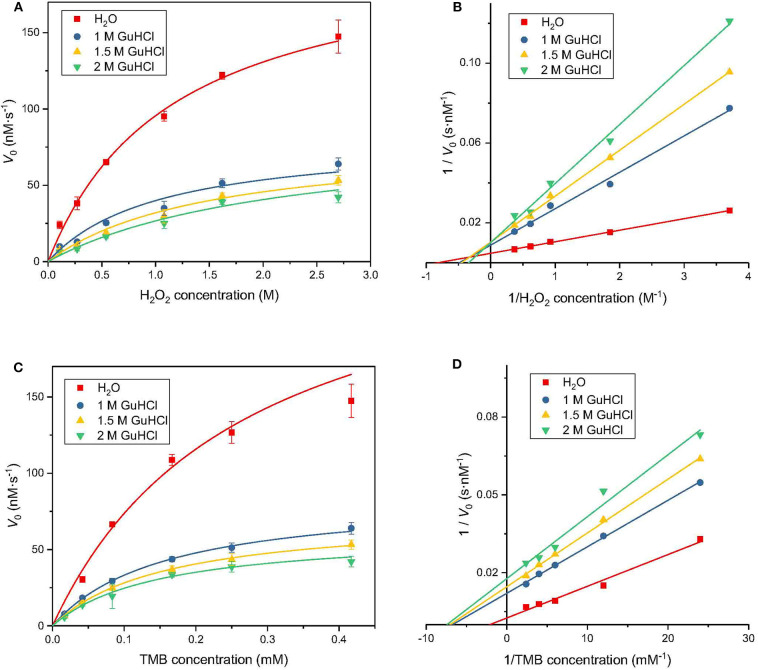
Inhibition kinetics analysis of IONzyme at different GuHCl concentrations. Conditions are shown in Figure 1, except that *K*_m_, *V*_max_, *k*_cat_, and *k*_cat_/*K*_m_ were analyzed at different H_2_O_2_ concentrations **(A)**. The Lineweaver-Burk plot of the data from **(A)** is shown in **(B)**. The data for different concentrations of TMB are shown in **(C)** and the Lineweaver-Burk plot of the data from **(C)** are in **(D)**. Data from 3 separate experiments (*n* ≥ 8). Data are the mean ± SD.

To investigate the mechanism of the interaction between GuHCl and IONzyme, we performed inhibition kinetic analysis. First, H_2_O_2_ was titrated at several fixed concentrations (1, 1.5, and 2 M) of GuHCl and at a fixed concentration of TMB. The results showed that the GuHCl-IONzyme interaction fit the Michaelis–Menten model. As shown in Figures 3A,B, the lines for GuHCl-treated samples intersected on the Y axis, suggesting that GuHCl is a competitive inhibitor of IONzyme against the substrate H_2_O_2_. Second, similar experiments were carried out with different concentrations of TMB and fixed concentrations of H_2_O_2_. During this time, the lines for GuHCl-treated groups intersected on the X axis, indicating that GuHCl is a non-competitive inhibitor of IONzyme with TMB as a substrate (Figures 3C,D). For both experiments, the lines of the control samples (without the addition of GuHCl) deviated somewhat from the intersection point compared to other lines.

The data were fitted to the Michaelis–Menten model to obtain the parameters, which are presented in Table 1. When using H_2_O_2_ as a substrate, Vmax is stable with increasing inhibitor concentration; that is, the effect of GuHCl can be eliminated by increasing the concentration of H_2_O_2_ in the reaction system. However, the value of Km gradually increased, i.e., with increasing inhibitor concentration, the affinity between IONzyme and H_2_O_2_ decreased, and these changes were consistent with the characteristics of competitive inhibitors. When TMB is used as a substrate, with increasing inhibitor concentration, Vmax decreases while Km remains unchanged, which is consistent with the characteristics of non-competitive inhibition. After the addition of 1 M GuHCl, the catalytic efficiency of IONzyme on TMB and H_2_O_2_ decreased by 46 and 56%, respectively, and both decreased further as the concentration of GuHCl was increased.

**Table 1:** Comparison of the kinetic parameters of Fe_3_O_4_ IONzymes in H_2_O or GuHCl.

	**[*E*] (M)**	**Substrate**	***K*_**M**_ (mM)**	***V*_**MAX**_ (M s^**−1**^)**	***k*_**CAT**_ (s^**−1**^)**	***k*_**CAT**_/*K*_**M**_ (s^**−1**^ M^**−1**^)**
IONzyme (in water)	4.2 × 10^−12^	TMB	0.267	2.70 × 10^−7^	6.43 × 10^4^	2.41 × 10^8^
	4.2 × 10^−12^	H_2_O_2_	1,165	2.07 × 10^−7^	4.93 × 10^4^	4.23 × 10^4^
IONzyme (GuHCl- 1 M)	4.2 × 10^−12^	TMB	0.157	8.52 × 10^−8^	2.03 × 10^4^	1.29 × 10^8^
	4.2 × 10^−12^	H_2_O_2_	1,033	8.12 × 10^−8^	1.93 × 10^4^	1.87 × 10^4^
IONzyme (GuHCl- 1.5 M)	4.2 × 10^−12^	TMB	0.158	7.26 × 10^−8^	1.73 × 10^4^	1.09 × 10^8^
	4.2 × 10^−12^	H_2_O_2_	1,496	7.98 × 10^−8^	1.90 × 10^4^	1.27 × 10^4^
IONzyme (GuHCl- 2 M)	4.2 × 10^−12^	TMB	0.148	6.12 × 10^−8^	1.46 × 10^4^	9.86 × 10^7^
	4.2 × 10^−12^	H_2_O_2_	2,200	8.52 × 10^−8^	2.03 × 10^4^	9.23 × 10^3^

*K_m_, the apparent affinity; [E] is the IONzyme concentration, K_m_ is the Michaelis constant, V_max_ is the maximal reaction velocity and k_cat_ is the catalytic constant, where k_cat_ = V_max_/[E]. k_cat_/K_m_ reflects the catalytic efficiency*.

### Inactivation May Not Depend on GuHCl-Induced IONzyme Aggregation

**Figure 4:**
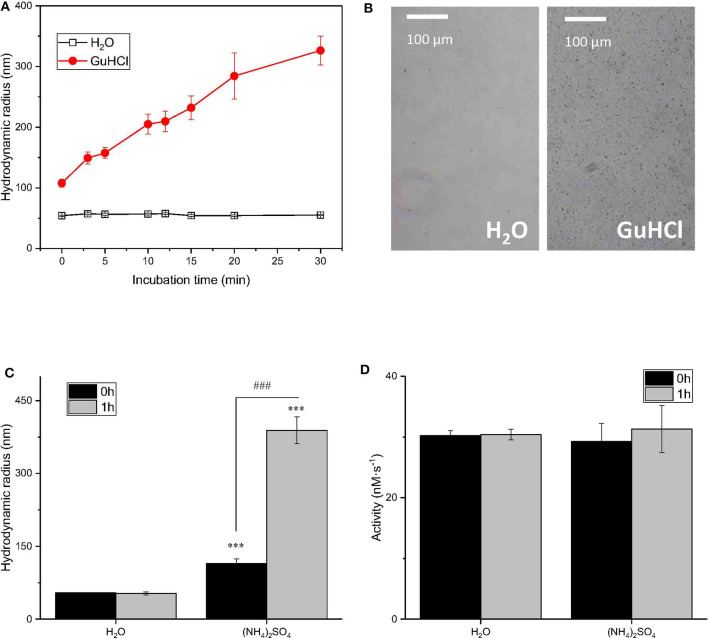
The relationship between the IONzyme peroxidase-like activity and the radius. Dynamic light scattering was employed to measure the hydrodynamic radii of IONzymes. Time course of IONzyme hydrodynamic radii in H_2_O or 2 M GuHCl **(A)** IONzyme aggregation was observed under a microscope after incubation the Nanozyme (0.91 μg/100 μl; concentration of Nanozyme in the Nanozyme-GuHCl incubation mixture before adding the reaction buffer) in 0.2 M GuHCl for 24 h **(B)** Comparison of IONzyme hydrodynamic radius **(C)** and peroxidase-like activity **(D)** in H_2_O or 0.5 M (NH_4_)_2_SO_4_ with 0 or 1 h pre-incubation time. ***P < 0.001 (vs. H_2_O−0 h). ^###^*P* < 0.001 (vs, (NH_4_)_2_SO_4_−0 h).

To determine whether the reported IONzyme aggregation was involved in the GuHCl-induced inactivation of IONzyme, in addition to the competitive interaction of GuHCl with substrate H_2_O_2_, the progressive aggregation of IONzymes was recorded during incubation with 2 M GuHCl for 30 min by dynamic light scattering. The size was represented by the hydrodynamic radius. As shown in Figure 4A, the size of IONzymes with water as a control did not significantly change during the incubation. However, the IONzymes with GuHCl were markedly larger than the control at 0 h and continued to increase with time, indicating that aggregates appear immediately upon the addition of GuHCl and continue growing. On the other hand, the activity of IONzymes, as mentioned previously, dropped immediately after IONzymes were mixed with GuHCl and remained stable at an inhibited level (35–55% of control) during the 30 min incubation (Figure 1D). Thus, the growth of aggregates did not enhance the inactivation. Since the activity of IONzymes was not changed at GuHCl concentrations >0.4 M, we determined whether IONzyme aggregation was induced at a low concentration of GuHCl (0.2 M) The results distinctly showed aggregation of IONzyme in 0.2 M GuHCl after 24 h, even using a μm-scale microscope (Figure 4B).

To demonstrate that aggregation did not affect the function of IONzyme, we employed 1 M (NH_4_)_2_SO_4_, another commonly applied inducer for protein aggregation, to determine whether it could mimic the effects of GuHCl. As shown in Figures 4C,D, the size of particles did not significantly change after 1 h of incubation in water (with radii of 54.18 ± 0.86 nm at 0 h and 53.02 ± 3.29 nm at 1 h) but was distinctly enlarged in (NH_4_)_2_SO_4_ with either no or 1 h pre-incubation, and longer pre-incubation times gave larger aggregates (with radii of 114.80 ± 9.37 nm at 0 h and 388.87 ± 27.56 nm at 1 h). Moreover, the aggregated samples with different sizes show similar activities. These data indicate that IONzyme aggregation, which was also induced by 1 M (NH_4_)_2_SO_4_,, does not affect the activity of IONzymes. Therefore, the GuHCl-IONzyme interaction definitely induces incidental IONzyme aggregation, but the aggregation may not contribute to GuHCl-induced IONzyme inactivation under our experimental conditions.

### Interaction Between GuHCl and Iron Atom Correlates With the Inactivation of IONzymes

**Figure 5:**
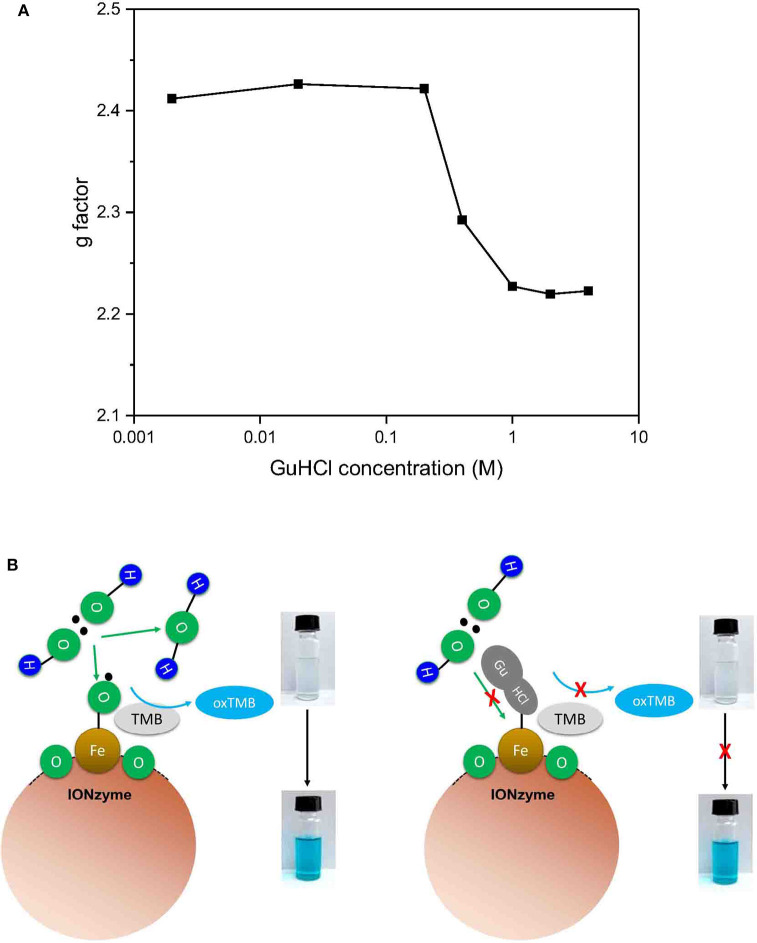
Mechanism of the peroxidase reaction catalyzed by IONzyme. Electron spin resonance spectroscopy was used to analyse the interaction between IONzyme and GuHCl in sodium acetate buffer. The change in the g factors of IONzymes in different concentrations of GuHCl **(A)**. A putative Scheme of IONzyme catalytic reaction **(B)**.

To check whether GuHCl binds to the iron atom in the interaction between GuHCl and IONzyme, we applied ESR spectroscopy to analyse the unpaired electrons in the IONzymes, which could reflect the character of the iron atom (Hagen, 2008). We mixed IONzyme with GuHCl in NaAC buffer (pH = 4.5) and measured the ESR spectrum at room temperature (Figure S2). As shown in Figure 5A, the g-factor value could be observed. The value of the g-factor was approximately 2.4 in the control and did not show a significant change in GuHCl at concentrations lower than 0.2 M. Then, it decreased quickly until the concentration of GuHCl reached 2 M and remained slightly >2.2. The change in the g-factor value with increasing GuHCl level shows a similar pattern to the inactivation of IONzymes under the same experimental conditions (Figure 1C, Figure S1). These results suggest that the activity changes in IONzyme are due to the interaction of IONzyme with GuHCl. GuHCl binds to the iron atom and affects the electron density of iron, inducing IONzyme inactivation. In addition, the inactivation level could be adjusted by GuHCl at concentrations between 0.4 and 2 M.

Based on the above experimental results, we established a model for IONzyme catalyzed reaction (Figure 5B). Since GuHCl binds to the iron atom on the IONzyme, and is a competitive inhibitor for H_2_O_2_, we conclude that H_2_O_2_ also binds the iron atom on the IONzyme during the reaction. GuHCl binds to the IONzyme non-competitively with TMB, so TMB binds to the oxygen atoms on the IONzyme.

## Discussion

In the current study, we employed GuHCl to investigate whether IONzyme has the characteristic inactivation property of a natural enzyme. The results show that GuHCl induces IONzyme aggregation and inhibits the representative peroxidase-like activity of IONzyme in a concentration- and treatment time-dependent manner. However, the aggregation process appears to be unrelated to inactivation; instead, GuHCl acts as a reversible inhibitor showing typical kinetic competition with substrate H_2_O_2_. ESR spectrum analysis revealed that the interaction of GuHCl with IONzyme induces changes in the unpaired electrons in the iron atom correlated with the inactivation of IONzyme. Our study revealed a reversible inhibition property of IONzyme mimicking that of protein enzymes and provided another way to interpret the catalytic mechanism and control nanozyme activity.

Similar to the inactivation and reactivation of a protein enzyme, the reversibility of the inactivation of IONzyme in the presence of GuHCl was also observed under our experimental conditions. To indicate this characteristic inactivation, we applied GuHCl and developed a procedure to affect the peroxidase-like activity of IONzymes. Although proteins undergo denaturation and renaturation, most proteins cannot be completely reactivated after inactivation by GuHCl denaturation; examples include creatine kinase (CK) (Zhou et al., 1993) and D-glyceraldehyde-3-phosphate dehydrogenase (GAPDH) (Liang et al., 1990). Proteins are characterized as “fragile” and are much more vulnerable to denaturants than IONzymes. The easy inactivation and reactivation of IONzyme suggest a convenient way to regulate its function by adjusting the concentration of GuHCl, which may facilitate the application of IONzyme.

GuHCl is widely used as a denaturant of authentic enzymes. As described previously (Zhang et al., 1993), protein inactivation and aggregation feature in “a fast inactivation followed by a slow conformational change:” that is, while the protein conformation undergoes little change, the enzymatic activity will decrease markedly (Xiao et al., 1993). This phenomenon is explained by the concept that the active site is much more flexible than the conformation of the molecule as a whole (Tsou, 1998). Therefore, the active site is vulnerable to disturbance by the denaturant GuHCl, and inactivation occurs before the overall protein molecular conformation changes. The denaturant GuHCl is regarded as a conformational change reagent but does not bind to the essential catalytic groups (Zhou et al., 1993). Now, we can see that GuHCl induces fast inactivation followed by a slow conformational (aggregation) change in IONzyme and acts as an inhibitor in addition to the common role of denaturant. On the other hand, the role of inhibitor was supported by the enzymatic kinetics assay. However, the straight line of the control sample (without the addition of GuHCl) in the reaction of GuHCl-treated IONzyme with different concentrations of H_2_O_2_ was somewhat decentralized in the double reciprocal plot. We suspect that there might be some other interaction between IONzymes and GuHCl.

In our experiment setup, the inhibition effect can be observed only when the concentration of GuHCl exceed 0.4 M, which is a fairly high concentration. GuHCl-induced aggregation could be observed in low-concentration GuHCl (0.2 M, Figure 4B). High-concentration (>0.4 M) GuHCl-induced quick reduction in IONzyme activity occurred before the aggregation is visible. Therefore, the threshold concentration of the GuHCl-induced inhibition cannot be fully explained by aggregation. Given that the activity of IONzyme in 1 M (NH_4_)_2_SO_4_ is the same as that in water, thus we can exclude the concern that the observed activity variation is due to the change of ionic strength and further support the conclusion that the effect is aggregation in-dependent. As mentioned above, GuHCl competes with substrate H_2_O_2_ on binding with the IONzyme. We hypothesized that the competence of GuHCl in competing with H_2_O_2_ on IONzyme binding is concentration-dependnet. In low GuHCl concentration (<0.2 M), IONzyme prior to bind H_2_O_2_ rather than GuHCl, which is support by the GuHCl concentration-dependent g-factor changes of the IONzyme in the ESR assay, suggesting a concentration-dependent GuHCl/nanozymes interaction ratio. Our result suggests that a novel interaction of GuHCl with the essential catalytic groups *in situ* at the active site occurred during GuHCl denaturation. Thus, clarification of the nanozyme characteristics helps us to reveal the reaction mechanisms not only of nanozyme but also of natural protein enzymes.

GuHCl, as an inhibitor, contains a guanidyl group and a chloride leading to the inactivation of IONzyme. The competitive manner between GuHCl and H_2_O_2_ exhibits both of them bind to iron atom in IONzyme. TMB probably binds to the oxygen of Fe_3_O_4_ because of the non-competitive reaction with GuHCl. Liu and colleagues described the inhibition of the enzymatic-like activity of gold nanoparticles in the presence of halide ions (Liu et al., 2017). They found that halide ions can switch the activity of gold nanozymes by Au-X interactions. Their results indicated that chloride may play an important role in the reaction with IONzyme. However, GuHCl can also be used in the inactivation of other nanozymes, although our work has mainly focused on IONzyme.

In summary, the denaturant GuHCl induces IONzyme aggregation and inhibits its representative peroxidase-like activity. The inactivation results mainly from the role of GuHCl as an inhibitor associated with the iron atom in IONzyme, instead of a denaturant related to conformation changes such as aggregation. This work demonstrates the reversible inhibition of IONzyme, mimicking that of protein enzymes, and provides an additional way to control nanozyme activity. It reveals the catalytic mechanism that H_2_O_2_ binds to the iron atom and TMB binds to oxygen atom in the IONzyme.

## Data Availability Statement

All datasets generated for this study are included in the article/Supplementary Material.

## Author Contributions

JY performed the experiments and wrote the manuscript. WM performed major parts of the experiments and wrote the manuscript. YL provided some ideas, discussed, and wrote the manuscript. YW and LG provided some ideas. RH designed, supervised, and wrote the manuscript.

## Conflict of Interest

The authors declare that the research was conducted in the absence of any commercial or financial relationships that could be construed as a potential conflict of interest. The reviewer XQ declared a shared affiliation, though no other collaboration, with the authors WM, JY, YL, YW, and RH.

## Supplementary Material

The Supporting Information is available free of charge on the ACS Publications website at doi: 10.3389/fchem.2020.00491, including the long-term effect of GuHCl on the activity of IONzyme and the ESR spectra of IONzymes mixed with different concentrations of GuHCl.
