# Supplementary material for: Reversible Inhibition of Iron Oxide Nanozyme by Guanidine Chloride
Source: Front Chem. 2020 Jun 11;8:491. doi: 10.3389/fchem.2020.00491 (PMC7301555; doi:10.3389/fchem.2020.00491)
Supplement: Supplementary file 1 [file Data_Sheet_1.pdf]

Supplementary Material

Supplementary Figure 1:

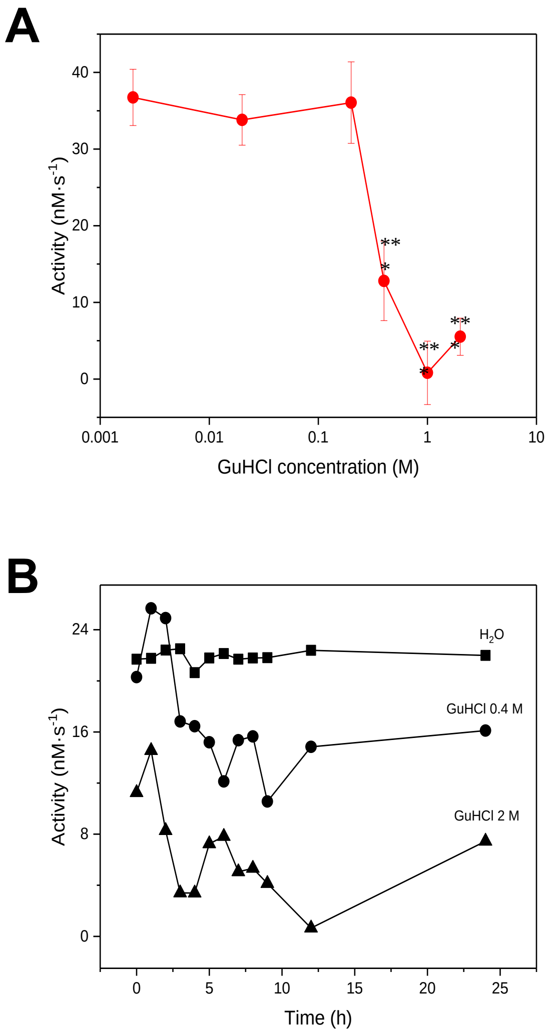

Figure S1: The long-term effect of salts on the activity of IONzyme. The concentration-dependent deactivation of IONzyme by 24 h incubation in GuHCl, indicating that the threshold of the GuHCl effect is not time dependent but instead that the concentration of GuHCl is the essential factor for the inhibition of IONzyme activity (n = 12) (panel A). Changes in the IONzyme peroxidase-like activity incubated with 2.0 M GuHCl at different times (0 – 24 h) (n = 18) (panel B). Data are presented as the mean ± SD. ***, P < 0.001.

Supplementary Figure 2:

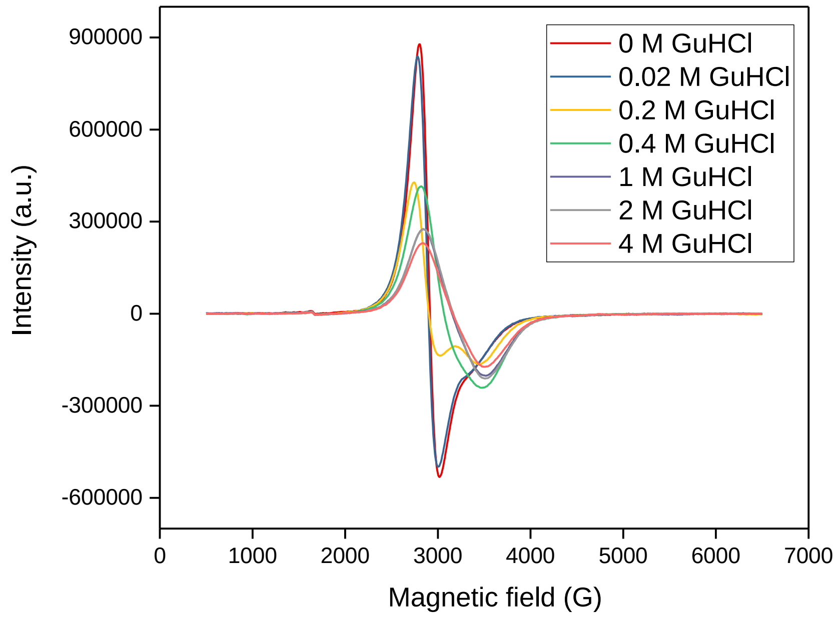

Figure S2: ESR spectra of IONzymes mixed with different concentrations of GuHCl. All ESR measurements were carried out using a Bruker A300-10/12 ESR spectrometer (Billerica, MA) at room temperature with 20 mW microwave power. The modulation field was 1G, and the scan range was 6000 G.

Supplementary Figure 3:

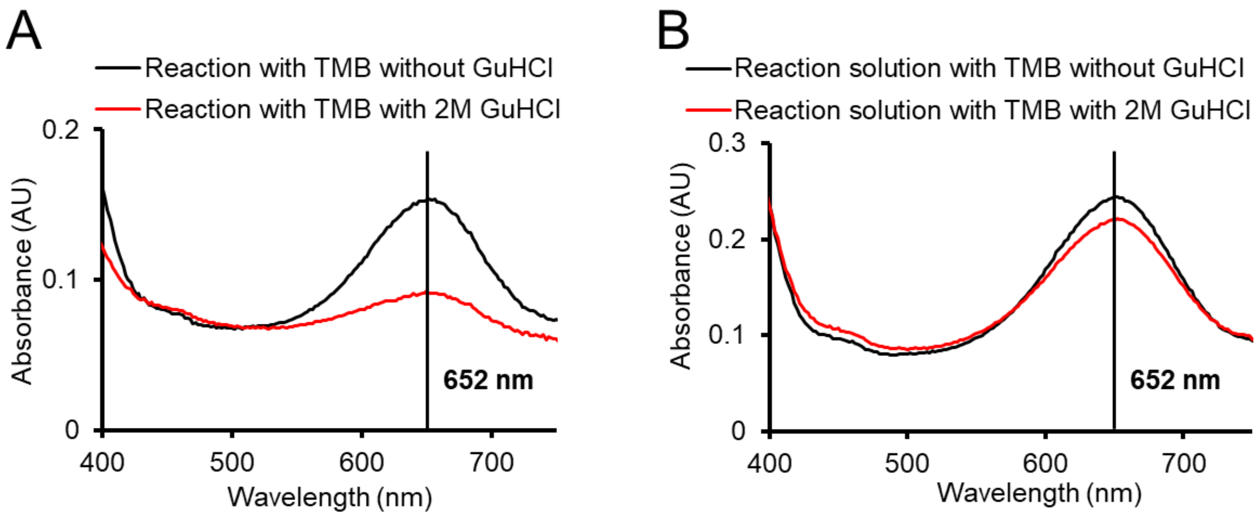

Figure S3: The UV/Vis absorption spectra of reaction solutions containing TMB with or without GuHCl. (A) The reaction was conducted with or without the presence of GuHCl as described in Figure 1. The UV/Vis absorption spectra (400-750 nm) of the reaction solutions after 5 min incubation was measured by a Microplate spectrophotometer (Molecular Devices, Sunnyvale, CA, USA). The absorption peaked around 652 nm and dramatically decreased with the presence of GuHCl. **(B)** The reaction was conducted for 20 min without the presence of GuHCl. Water or 2M GuHCl was added into the blue-coloured reaction solution. The UV/Vis absorption spectra of the water-diluted and GuHCl-mixed reaction solutions were measured immediately. Compared with the results in panel A, the GuHCl did not significantly change the colour of the reaction solution (UV/Vis absorption spectra).

Materials and Methods

Synthesis of IONzymes

In a typical procedure, 0.82 g $FeCl_3$ was dissolved in 40 mL ethylene glycol with vigorous stirring. When the solution became clear, 3.6 g NaAc was added with continuous stirring for

30 min. The mixture was then transferred into a 50 mL Teflon-lined stainless-steel autoclave and reacted at 200 °C for 12 h. After the reaction was completed, the autoclave was cooled to room temperature. The products in a black colour were collected, rinsed with ethanol for several times, and dried at 60 °C.
